# Amiloride Promotes Oligodendrocyte Survival and Remyelination after Spinal Cord Injury in Rats

**DOI:** 10.3390/jcm7030046

**Published:** 2018-03-05

**Authors:** Takeshi Imai, Hiroyuki Katoh, Kaori Suyama, Masahiro Kuroiwa, Sho Yanagisawa, Masahiko Watanabe

**Affiliations:** Department of Orthopaedic Surgery, Surgical Science Tokai University School of Medicine 143 Shimokasuya, Isehara, Kanagawa 259-1193, Japan; tokai-takeshi@tokai-u.jp (T.I.); hero@tokai-u.jp (H.K.); suyama@is.icc.u-tokai.ac.jp (K.S.); kuroiwa@is.icc.u-tokai.ac.jp (M.K.); syngsw@tsc.u-tokai.ac.jp (S.Y.)

**Keywords:** spinal cord injury, amiloride, oligodendrocyte precursor cell

## Abstract

After spinal cord injury (SCI), secondary injury results in an expanding area of glial cell apoptosis. Oligodendrocyte precursor cells (OPCs) actively proliferate after SCI, but many of these cells undergo apoptosis. One of the factors that exacerbates secondary injury is endoplasmic reticulum (ER) stress. In this study, we tested the effects of amiloride treatment on the fate of OPCs during secondary injury in rats. Amiloride is an FDA-approved diuretic for treating hypertension, which in rats enhances ER stress response and suppresses the apoptosis of glial cells after SCI. A severe contusive SCI was induced in Sprague-Dawley rats using an infinite horizon (IH)-impactor (200 kdyne). Beginning 24 h after SCI, 10 mg/kg of amiloride or phosphate buffered saline (PBS) was intraperitoneally administered daily for a period of 14 days. At 7, 14, 28, and 56 days after SCI, animals were subsequently euthanized in order to analyze the injured spinal cord. We labeled proliferating OPCs and demonstrated that amiloride treatment led to greater numbers of OPCs and oligodendrocytes in the injured spinal cord. Increased myelin basic protein (MBP) expression levels were observed, suggesting that increased numbers of mature oligodendrocytes led to improved remyelination, significantly improving motor function recovery.

## 1. Introduction

Spinal cord injury (SCI) is a permanently debilitating injury that lowers the quality of life, not only for the patient, but also their family members. Worldwide, an estimated 2.5 million people live with SCI, with more than 130,000 new injuries reported annually [1]. There is a global effort to decrease the number of disabilities caused by SCI and improve patient recovery by employing strategies such as medication, cell transplantation, and rehabilitation. However, an effective treatment remains elusive.

The pathological process of SCI can be divided into; the primary injury caused by external forces causing physical tissue disruption; and secondary injury, in which biochemical and vascular factors cause delayed damage to tissue that survived the initial injury. During secondary injury, there is widespread apoptosis of neurons, astrocytes, oligodendrocytes, and microglia [2,3]. The apoptosis of oligodendrocytes, which begins hours after injury and continues for weeks, is especially detrimental, and contributes to the demyelination of neurons that survived the primary injury [4,5,6]. Although oligodendrocyte precursor cells (OPCs) proliferate at the periphery of the lesion site and remyelinate spinal axons, this process is limited because a large percentage of OPCs succumb to apoptosis before differentiating into mature oligodendrocytes [7,8]. Therefore, we have been searching for a treatment that inhibits the apoptosis of OPCs in the injured spinal cord, which in turn would reduce the expansion of secondary injury, and improve function through increased remyelination.

One of the factors implicated in OPC apoptosis is endoplasmic reticulum (ER) stress, which occurs due to the accumulation of misfolded proteins in the ER [9,10]. Unfolded or misfolded proteins are a common occurrence in cellular maintenance, and are usually handled by an intricately choreographed process, called the ‘ER stress response’ or ‘unfolded protein response (UPR)’. The ER stress response aims to reduce ER stress and restore equilibrium by slowing protein translation, degrading misfolded proteins, and increasing molecular chaperones that fold proteins, such as glucose-regulated protein 78 (GRP78). However, when ER stress overwhelms the capacity of the UPR to restore equilibrium, apoptosis is induced through multiple pathways, including CCAAT/enhancer-binding protein (C/EBP) homologous transcription factor protein (CHOP), caspase 12, and Jun kinase.

Previous studies have demonstrated that SCI induces an increase of ER stress, with different ER stress responses being observed according to neural cell type [10]. Furthermore, it was shown that lowering the ER stress response of OPCs leads to increased apoptosis during the secondary injury phase of SCI. Studies have also demonstrated that the administration of amiloride, which enhances the ER stress response, decreased OPC apoptosis [11,12]. In our previous study by Kuroiwa, et al. [9], we demonstrated that amiloride administration increased GRP78 expression and decreased CHOP expression within the spinal cord, which decreased neural cell apoptosis and led to an increase of OPCs in the injured spinal cord. The group treated with amiloride had improved motor function compared to the control group up to 28 days after injury. In this follow-up study, we use the same amiloride treatment and study the tested animals up to 56 days after injury to examine the following points: (1) does the increase in OPCs lead to increased oligodendrocytes; (2) does the increase in oligodendrocytes lead to improved remyelination; and (3) is there further improvement in motor function after 28 days, and what effect does amiloride treatment have on allodynia after SCI?

## 2. Experimental Section

### 2.1. SCI Model

Female Sprague-Dawley (SD) rats (8–10 weeks old and weighing 280–320 g) were obtained from CLEA Japan, Inc. (Kanagawa, Japan).

Surgical procedures were performed under aseptic conditions, and the rats were anesthetized by inhaling 4% isoflurane. Laminectomy of the 10th thoracic vertebra was performed to expose the dura mater. A severe spinal cord contusion injury was created using an Infinite Horizon spinal cord impactor device (IH impactor; Precision Systems & Instrumentation, Lexington, KY, USA) with a force of 200 kdyne (2 mN) and a dwell time of zero seconds. To treat the bladder dysfunction that commonly occurs after SCI, abdominal massages were performed twice a day until urinary function was regained.

All experimental procedures were performed in accordance with the National Institute of Health Guidelines for the Care and Use of Laboratory Animals, and were approved by the Animal Experimentation Committee of Tokai University School of Medicine (21591907).

### 2.2. Administration of Amiloride

The injured rats were randomly divided into two groups: an amiloride group, and a phosphate buffered saline (PBS) control group. At 24 h after injury, and every 24 h thereafter for 14 days, the amiloride group received via intraperitoneal administration 10 mg/kg of amiloride (amiloride hydrochloride hydrate, A7410; Sigma-Aldrich, St. Louis, MO, USA) in accordance with a protocol used for a mouse multiple sclerosis model [13]. The drug dosage was calculated from the body weight of each rat, which was measured daily. The PBS group received intraperitoneal administrations of PBS using the same protocol.

At 24 h after injury, and every 12 h thereafter for three days, both groups received intraperitoneal administrations of 50 mg/kg of 5-bromo-2-deoxyuridine (BrdU, Sigma-Aldrich, B5002, St. Louis, MO, USA) in order to trace the cells proliferating after SCI.

### 2.3. Evaluation of Hindlimb Function

#### 2.3.1. Basso, Beattie, Breanahan Locomotor Rating Scale (BBB) Scores

Hindlimb motor function after SCI was assessed weekly using the BBB scale [14], which is an open-field locomotor test for rats. Locomotor behavior was evaluated immediately before injury and at approximately the same time each week for the following eight weeks after injury (*n* = 5 per group).

#### 2.3.2. Dynamic Plantar Esthesiometer

To assess changes in sensation or the development of mechanical allodynia, sensitivity to tactile stimulation was assessed using the dynamic plantar esthesiometer (DPA, UGO BASILE, catalog#37450, Monvalle VA, Italy), which is an automated version of the von Frey hair assessment. Rats were individually placed in a small, enclosed testing arena with a wire mesh floor for 5 min. The DPA device was positioned beneath the rats so that the filament always applied pressure to the center of the sole of the foot, which is defined as the midpoint between both the medial–lateral and the anterior–posterior axes of the foot. If an animal’s movement caused a different part of the foot to be stimulated, the data was disregarded and another attempt was made. When initiated, the device raised the filament to touch the plantar surface of the foot and progressively increased the force until the animal withdrew its foot, or until it reached a maximum force of 50 g. The DPA automatically recorded the force at which the foot was withdrawn as well as the withdrawal latency. Each paw was tested five times per session. This testing was performed prior to surgery, and once a week for eight weeks after SCI (*n* = 5 per group).

### 2.4. Immunohistochemistry

At 7, 14, 28, and 56 days after injury, intracardial perfusion fixation was performed using 0.1 M PBS and 2% paraformaldehyde (PFA) whilst the test subjects were under a 4% isoflurane anesthesia. After fixation, the spinal cord was excised and post-fixed in 2% PFA in 0.1 M PBS for two days at a temperature of 4 °C, and was cryoprotected by soaking it in 15% sucrose for five days. The epicenter was defined as the 2 mm-width of the tip of the IH impactor, and the spinal cord was transected into 3 mm segments: a center segment containing the lesion epicenter, and segments that were both rostral and caudal to the epicenter. Each segment was embedded in an optimal cutting temperature (OCT) compound (Sakura Finetek, Tokyo, Japan), frozen in liquid nitrogen, and sectioned at a thickness of 10 µm using a cryostat microtome (CM3050S, Leica Biosystems, Cincinnati, OH, USA). From this epicenter, sections of the spinal cord were selected from an area approximately 5 mm caudal to the epicenter and underwent immunohistochemical analyses.

The sections were washed three times (5 min each) with PBS, incubated for 10 min in 2N HCl for antigen activation, and rinsed in a boric acid buffer for neutralization. The sections were then washed four times with PBS, blocked for 60 min in PBS with a 5% normal goat serum at a temperature of 24 °C, and incubated overnight at 4 °C with anti-BrdU antibodies (GE Healthcare, anti-mouse; RPN202). After washing with PBS, the sections were incubated in the dark for 60 min at 24 °C, with the respective fluorescent secondary antibodies (Alexa Fluor594, anti-mouse; abcam, ab150116, 1:1000). In the dark, the sections were washed four times and incubated overnight at 4 °C with anti-NG2 (rabbit; a marker for OPCs; Millipore, Bedford, MA, USA, A1B5320, 1:500), or anti-adenomatous polyposis coli (APC) antibodies (rabbit; a marker for mature oligodendrocyte; abcam, ab15270, 1:500). The sections were washed again with PBS and incubated for 60 min at 24 °C with the respective fluorescent secondary antibodies (Alexa Fluor488, anti-Rabbit, abcam, ab150077, 1:1000). Subsequently, nuclei were stained using Vectashield with 4′,6-diamidino-2-phenylindole (DAPI) H-1500 (Vector Laboratories) and then mounted. Images were taken using a Nikon DS-Ri1 camera with mono 10-bit lenses (40 × objective Numerical Aperture (N.A.) = 0.6) on an Olympus IX70 fluorescence microscope and analyzed with Nikon NIS-elements Version 3.1 software (Nikon, Tokyo, Japan). The number of proliferating OPCs (NG2-positive cells) or mature oligodendrocytes (APC-positive cells) were quantified by counting the number of cells positive for both BrdU, and each cell marker within the entire cross-section of the dorsal funiculus; only cells with clearly recognizable nuclei and cytoplasm that had cytoplasmic staining that corresponded to each secondary antibody were scored as positive. A sample size of *n* = 5 rats per group and per time-point was used, and three sections were observed for each rat. Since a few immunohistochemical labels are not specific to a single cell type, we also relied on morphology to determine the cell type. For example, NG2-positive OPCs were distinguished from NG2-positive macrophages by morphology: OPCs are small cells with multiple processes, whereas macrophages are large, amoeboid cells without processes. Macrophages were not included in the NG2-positive cell counts.

### 2.5. Western Blot

At 7, 14, 28 and 56 days after injury, rats were anesthetized via the inhalation of 4% isoflurane, and subsequently 5 mm of the injured spinal cord (located 2.5 mm rostral and caudal to the epicenter) was microscopically dissected. The excised spinal cord tissue was immediately washed in ice-cold PBS, after which extracts were prepared using freshly formulated cell lysis buffer (50 mM Tris/HCl pH 7.4, 1 mM CaCl2, 0.5 mM phenylmethylsulfonyl fluoride (PMSF), 1% Nonidet-P (NP)-40) and sonicated using an Ultras Homogenizer VP-5x (Taitec, Saitama, Japan). All protein extraction procedures were performed on ice. These extracts were subjected to electrophoresis on a 15% sodium dodecyl sulfate polyacrylamide gel; 2.5 µg of protein solution was applied to each gel lane. After electrophoresis, the proteins were electrotransferred onto nitrocellulose membranes. The membranes were blocked with 3% bovine serum albumin in Tris-buffered saline with Tween-20 (TBST, 50 mM Tris, pH 7.6, 150 mM NaCl, 0.1% Tween-20), followed by incubation with anti-myelin basic protein (MBP, rabbit, abcam, ab40390, 1:8000) antibodies overnight at 4 °C. The membranes were washed for three hours in PBS containing 0.05% Tween-20 and incubated for 60 min at 24 °C with horseradish peroxidase (HRP)-linked anti-rabbit IgG (catalog #NA9340; GE Healthcare, Amersham, UK, 1: 12,000). Beta-actin was used as an internal control and was labeled with a mouse monoclonal anti-beta-actin antibody (Sigma-Aldrich, A5441, 1:1000). The proteins were labeled with Immobilon Western Chemiluminescent horseradish peroxidase (HRP) (Millipore, Bedford, MA, USA), and protein concentrations were quantified by densitometric scanning assays using the image analysis software CS Analyzer (Atto, Tokyo, Japan). The analysis was performed separately for each lane with a consistently sized designated area with background subtraction and normalization to actin. For each time-point, a sample size of *n* = 5 was used and the average was calculated for the group.

### 2.6. Toluidine Blue Staining and Electron Microscopy

At 28 days after injury, rats were anesthetized via the inhalation of 4% isoflurane, and intracardial perfusion fixation was performed with 2% PFA and 2% glutaraldehyde in 0.1 M PBS. After fixation, the spinal cord was excised 5 mm rostral to the epicenter and post-fixed in 2% osmium tetroxide in 0.1 M PBS. The samples were dehydrated in ethanol and embedded. Transverse ultrathin sections were cut with an ultramicrotome (LKB-2088) and examined by electron microscopy (JEM-1400, JEOL, Tokyo, Japan).

To quantify remyelination, a blinded technician was instructed to take 10 images from randomly selected areas on the periphery of the glial scar within the dorsal funiculus surrounding the epicenter of the lesion. The image files were randomized, and the primary investigator performed the quantification of myelination, which is express as G ratio = Myelin sheath thickness/axon diameter. Two ultrathin sections were used from each rat, and a sample size of *n* = 5 was used per group.

### 2.7. Statistical Analysis

The BBB scale scores were analyzed by two-way repeated-measures analysis of variance (ANOVA) with Tukey’s post hoc multiple comparison tests. Similarly, the results of the von Frey test were also analyzed by ANOVA with Tukey’s post-hoc multiple comparison tests. Data obtained through immunohistochemistry, MBP expression levels, as well as the ratio of myelin sheath thickness to the axon diameter were analyzed using Mann–Whitney *U* tests. Statistical significance was determined using IBM SPSS Statistics for Windows, Version 23.0 (IBM Corp., Armonk, NY, USA). Asterisks in figures indicate statistical significance (* *p* < 0.05, ** *p* < 0.01).

## 3. Results

### 3.1. Hindlimb Motor Function

**Figure 1:**
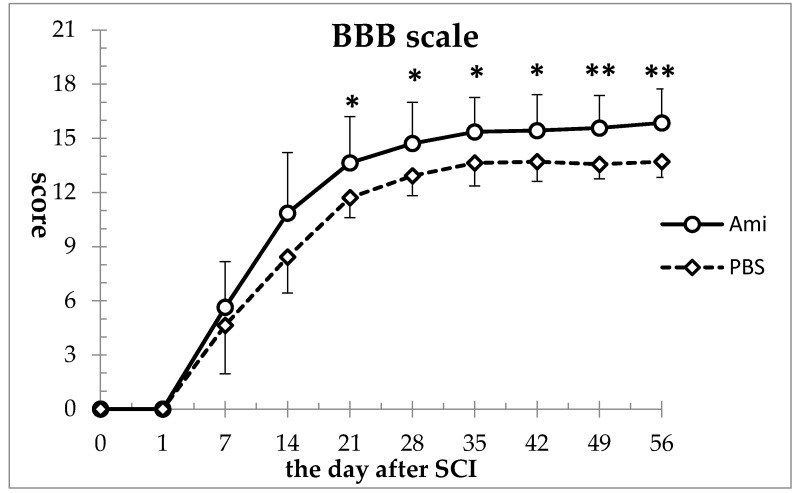
Amiloride improved motor function recovery after spinal cord injury (SCI). Basso, Beattie, Breanahan locomotor rating scale (BBB) scale scores were significantly higher in the amiloride group compared to the phosphate buffered saline (PBS) group from day 21 to day 56. Asterisks indicate significant difference between the amiloride group (Ami) and the PBS group (PBS) with two-way repeated measures analysis of variance (ANOVA), followed by Tukey’s multiple comparison test (* *p* < 0.05, ** *p* < 0.01, *n* = 5 per group, error bars are standard deviations).

In both the amiloride and the phosphate-buffered saline (PBS) groups, the BBB scale scores that were 21 before injury dropped to 0 after injury, and gradually improved thereafter. Compared to the PBS group, the amiloride group showed improved hindlimb motor function from as early as day 7 (amiloride group: 5.64 ± 2.62, PBS group: 4.64 ± 2.79), however the differences were not statistically significant at this time-point. The difference between the two groups reached statistically significant levels starting at day 21 (amiloride group: 13.64 ± 2.55, PBS group: 11.71 ± 1.10, *p* < 0.05), and this significant difference was maintained for the 56-day duration of the study (amiloride group: 15.85 ± 1.88, PBS group: 13.71 ± 0.88, *p* < 0.001) (Figure 1).

### 3.2. Hindlimb Sensory Function

**Figure 2:**
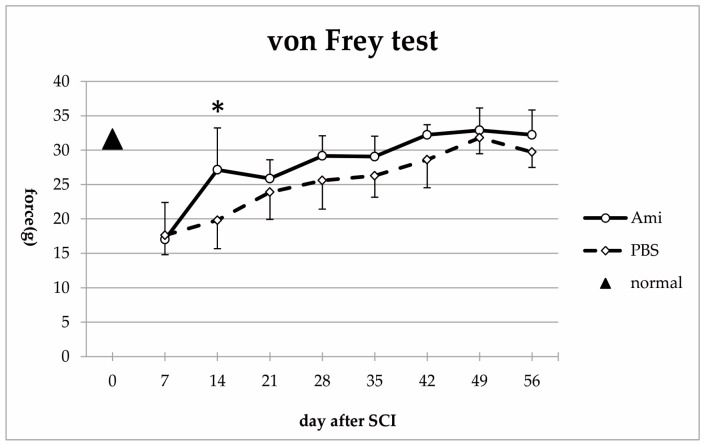
Compared to control rats (triangle, *n* =5), a sharp drop in the mean force required to elicit a paw withdrawal was observed in both groups after SCI, suggesting the development of allodynia. Amiloride treatment improved sensory function recovery after SCI, with the amiloride group registering a significantly higher force compared with the PBS group at day 14. The asterisk indicates a significant difference between the amiloride group (Ami) and the PBS group (PBS) with two-way repeated measures ANOVA, followed by Tukey’s multiple comparison test (* *p* < 0.05, *n* = 5 per group, error bars are standard deviations).

Before injury, the mean force required to generate paw withdrawal in the Von Frey test was 31.60 ± 3.44 g. After injury, there was a sharp decrease in the forces required to elicit a response in both the amiloride and the PBS groups (day 7; amiloride group: 17.00 ± 5.40 g, PBS group: 17.62 ± 2.82 g), indicating impaired sensory function in the form of allodynia. At day 14, the amiloride group showed a significant improvement compared to the PBS group (amiloride group: 27.14 ± 6.08 g, PBS group: 19.81 ± 4.12 g, *p* < 0.05). Thereafter, improvements in sensory function were observed in both groups, with the amiloride group having higher forces. However, the differences with the PBS group did not reach statistical significance (day 56; amiloride group: 32.33 ± 3.60 g, PBS group: 29.72 ± 2.24 g) (Figure 2).

### 3.3. Survival and Proliferation of Oligodendrocyte Precursor Cells (OPCs)

**Figure 3:**
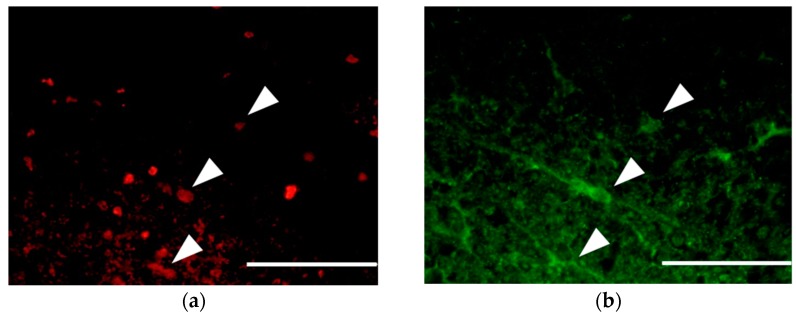

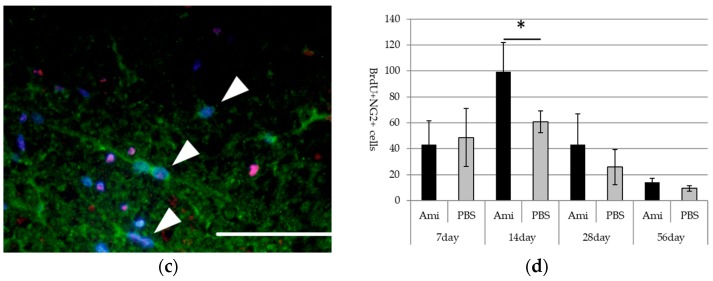
(**a**–**c**) Double immunostaining of the dorsal funiculus from the amiloride group at day 14 for BrdU (red) and NG2 (green) with 4′,6-diamidino-2-phenylindole (DAPI) nuclear counterstaining (blue). The arrowheads indicate the BrdU- and NG2-positive cells (scale bars 50 μm); (**d**) In both the amiloride and PBS groups, an increase of BrdU- and NG2-positive cells was observed at day 14, followed by a decrease to day 56. Significantly more BrdU- and NG2-positive cells were observed in the amiloride group compared to the PBS group on day 14. The asterisk indicates significant difference between the amiloride and PBS groups with a Mann–Whitney *U* test (* *p* < 0.05, *n* = 5 per group, error bars are standard deviations).

**Figure 4:**
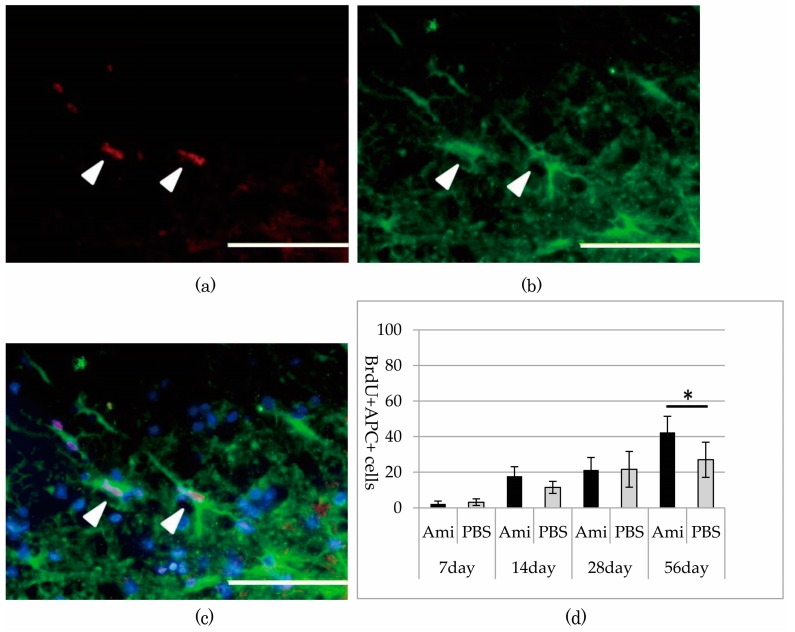
(**a**–**c**) Double immunostaining of the dorsal funiculus from the amiloride group at day 56 for BrdU (red) and adenomatous polyposis coli (APC) (green) with DAPI nuclear counterstaining (blue). The arrowheads indicate the BrdU- and APC-positive cells (scale bars 50 μm); (**d**) In both the amiloride and PBS groups, the number of BrdU- and APC-positive cells increased after SCI. Significantly more BrdU- and APC-positive cells were observed in the amiloride group than in the PBS group on day 56. The asterisk indicates significant difference between the amiloride and PBS groups with a Mann–Whitney *U* test (*p < 0.05, *n* = 5 per group, error bars are standard deviations).

Immunohistochemistry revealed BrdU-positive cells at the periphery of the glia scar in the dorsal funiculus, indicating that neural cells replicated at that location between 12 to 72 h after SCI. Specifically noting the BrdU-positive OPCs that proliferated during the period after SCI, the number of BrdU- and NG2-positive OPCs peaked in both the amiloride and PBS groups at day 14. However, the number of new OPCs was significantly higher in the amiloride group than in the PBS group (amiloride group: 99.25 ± 22.69, PBS group: 60.80 ± 8.33, per section, *p* < 0.05) (Figure 3). Thereafter, we observed a gradual increase in the number of BrdU- and APC-positive oligodendrocytes that differentiated from OPCs proliferating after SCI. The amiloride group had a significantly greater number of BrdU- and APC-positive oligodendrocytes at day 56 than the PBS group (amiloride group: 42.40 ± 9.07, PBS group: 27.00 ± 9.91, per section, *p* < 0.05) (Figure 4), indicating that the decrease in OPC apoptosis caused by amiloride treatment led to an increase in the number of oligodendrocytes that differentiated from these OPCs.

### 3.4. Remyelination

**Figure 5:**
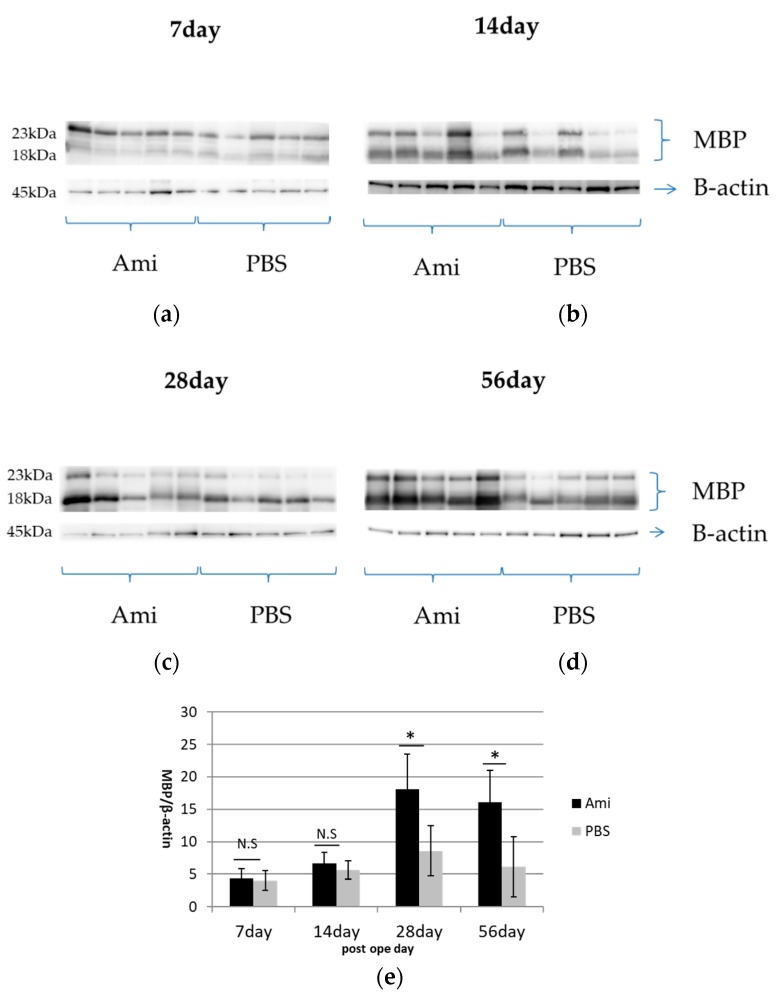
(**a**–**e**) Western blotting was performed using anti- myelin basic protein (MBP) (18, 23 kDa) and anti-b actin (45 kDa) antibodies. At days 28 and 56 after injury, MBP expression was significantly higher in the amiloride group compared to the PBS group. Asterisks indicate significant difference between the amiloride and PBS groups with a Mann–Whitney *U* test (* *p* < 0.05, *n* = 5 per group, error bars are standard deviations).

**Figure 6:**
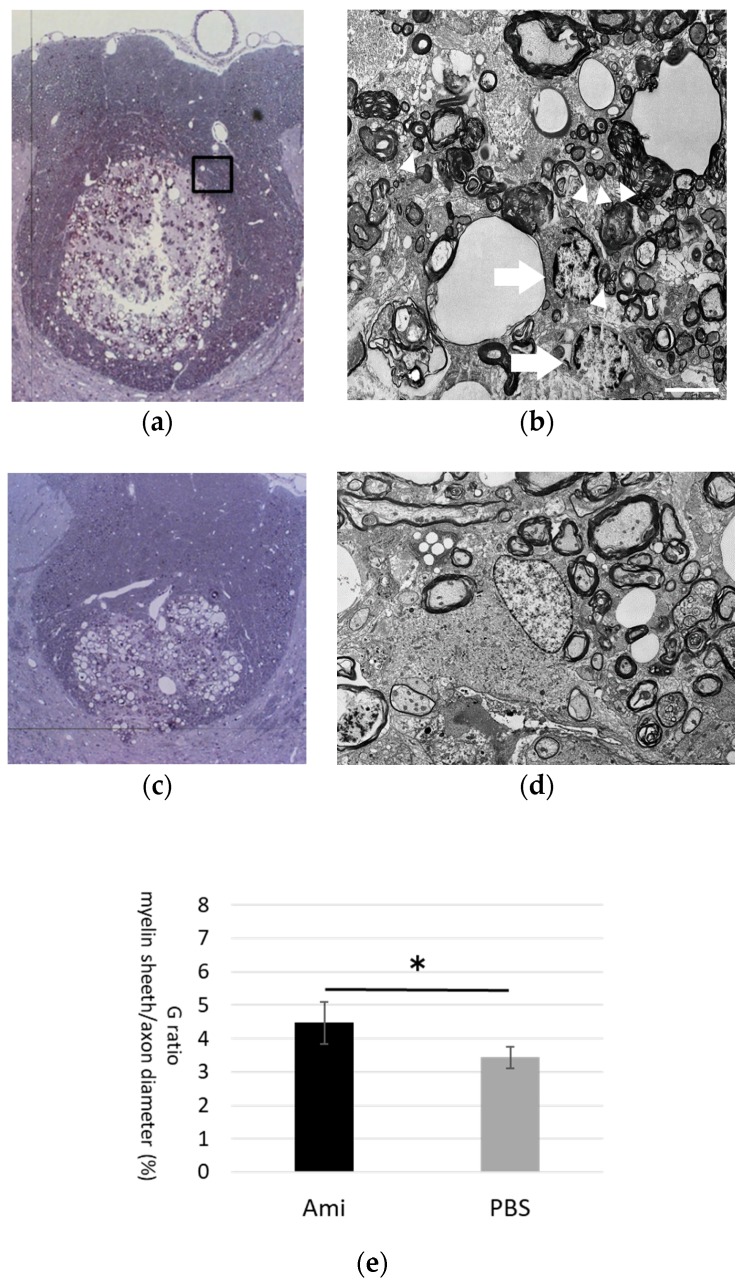
(**a**) Toluidine blue staining of the injured spinal cord from the amiloride group on day 28, with sections taken 7 mm rostral from the epicenter. Electron microscope analysis was conducted on the boxed glial scar area surrounding the epicenter of the lesion; (**b**) Electron microscopy revealed oligodendrocytes (arrows) near the axons (arrowheads) that had smaller diameters compared to axons distant from the injury site (scale bars 5 μm); (**c**,**d**) PBS group; (**e**) Myelin sheath thickness/axon diameter in the amiloride group was significantly higher than the PBS group at day 28. The asterisk indicates significant difference between the amiloride and PBS groups with a Mann–Whitney *U* test (* *p* < 0.05, error bars are standard deviations).

Western blot analyses were conducted to examine myelination by quantifying MBP expression. MBP expression, which was 21.58 in control rats (*n* = 6), decreased sharply in both the amiloride and PBS groups at day 7, and gradually increased thereafter up to day 56. The MBP expression was significantly higher in the amiloride group at days 28 and 56 after SCI (day 28, amiloride group: 18.08 ± 5.47, PBS group: 8.59 ± 3.90, *p* < 0.05; day 56, amiloride group: 16.08 ± 4.97, PBS group: 6.16 ± 4.65, *p* < 0.05) (Figure 5). The degree of remyelination was evaluated by electron microscopy. The average number of remyelinated fibers per EM field was significantly higher in the amiloride group compared to the control group (amiloride group: 346 ± 122, PBS group: 190 ± 65.5, *p* < 0.05). The average number of demyelinated fibers per EM field was higher in the amiloride group compared to the control group, but the difference was not significant (amiloride group: 670 ± 150, PBS group: 548 ± 71.3). Myelin thickness, evaluated as G-ratios, were significantly higher in the amiloride group compared to the PBS group at day 28 (amiloride group: 4.47 ± 0.63%, PBS group: 3.43 ± 0.33%, *p* < 0.05), suggesting that the increase in oligodendrocytes caused by amiloride treatment led to improved remyelination (Figure 6).

## 4. Discussion

### 4.1. The Apoptosis of Neural Cells during the Secondary Injury Phase of SCI

Following the primary injury phase of SCI, a collective phenomenon termed ‘secondary injury’ further damages the injured spinal cord. Over 25 distinct factors, including vascular (ischemia, edema), cellular (acidosis), and biochemical processes (inflammatory cytokines, increased glutamic acid, disruption of electrolyte balance) have been reported to occur during the secondary injury phase [6,15,16]. The major effect of secondary injury is the apoptosis of neurons, oligodendrocytes, microglia, and astrocytes, which has been shown to be triggered by an influx of calcium ions [17]. The disruption of the intracellular calcium ion balance activates catabolic enzymes, such as caspases and calpains, which degrade cellular proteins and disrupt the exoskeleton and membranes of the cell, leading to apoptosis [18]. Inflammatory cytokines, free radicals, and excitotoxicity have also been implicated in the apoptosis of cells distant from the epicenter of the injury [19,20].

The apoptosis of oligodendrocytes continues for several weeks after SCI and leads to the demyelination of axons that were spared from primary injury [4,5,6]. The demyelinated axons suffer from conduction delays and conduction blocks that severely impair the neural pathway between the brain and the distal spinal cord. OPCs are known to replicate at the periphery of the injury site, however a large percentage are lost to apoptosis without differentiating into mature oligodendrocytes [7,8]. Therefore, if it is possible to suppress the apoptosis of OPCs, the surviving OPCs may improve functional recovery by remyelinating bare axons.

### 4.2. ER-Stress-Induced Apoptosis

One of the pathways leading to apoptosis of OPCs is ER stress, which is responsible for the delayed cell death observed in multiple neurological conditions such as multiple sclerosis [13], Alzheimer’s disease, and Parkinson’s disease [21].

We have previously reported the involvement of ER stress in the apoptosis of neural cells during SCI. In an in vitro experiment to evaluate the cytoprotective function of GRP78 in the setting of increased ER stress, we engineered cultured glial cells to overexpress GRP78. When these cells were exposed to the ER-stress-inducing drug tunicamycin, the apoptosis of cells overexpressing GRP78 was significantly lower than that of control cells [22]. In an in vivo experiment, we compared the expression of ER-stress-regulating proteins GRP78 and CHOP in neurons, OPCs, oligodendrocytes, and astrocytes in a contusive SCI model. The expression of GRP78 was lower and the expression of CHOP was higher in OPCs when compared to other neural cells, suggesting that the lower tolerance of OPCs to ER stress could be involved in the apoptosis of OPCs and the ensuing disturbance of remyelination [23]. 

### 4.3. Enhancing the ER Stress Response with Amiloride

We next performed experiments to verify if the apoptosis of OPCs can be reduced by enhancing the ER stress response using amiloride, a Food and Drug Administration (FDA) -approved diuretic that has neuroprotective properties that inhibit acid-sensing ion channels, Na^+^/H^+^ exchangers, and Na^+^/Ca^2+^ exchangers [24]. Amiloride reduced the apoptosis of glial cells cultured under ER-stress-enhanced conditions [25], and is reported to have cytoprotective and ER-stress-modulating effects in numerous neurological diseases, such as brain ischemia [26], multiple sclerosis [13], Parkinson’s disease [21] and SCI [12]. We must also point out that the beneficial effects of amiloride may not be limited to its ER-stress-enhancing properties. After traumatic brain and SCI, persistent activation of voltage-sensitive sodium channels has been associated with cellular toxicity that leads to the degeneration of neural tissue. One of the most intensely studied drugs is riluzole, with studies concerning its therapeutic effects as a sodium channel blocker, but amiloride has also been shown to be neuroprotective because of its ability to block these ion channels.

In a recent experiment, we administered amiloride to a rat SCI model to examine its effect on ER stress and apoptosis. Compared to control animals, the rats that received amiloride administration had enhanced ER stress responses, with significantly increased expression of GRP78 and decreased expression of CHOP. The enhanced ER stress response led to a significant reduction in apoptosis and secondary injury expansion, and induced a significant increase in OPCs [9].

### 4.4. The Survival and Differentiation of OPCs

In a rat spinal cord chemical demyelination model using lysophatidylcholine, OPCs replicated and differentiated into oligodendrocytes [27]. BrdU cell-tracing experiments confirmed that the differentiated oligodendrocytes were descendants of OPCs that replicated after the chemical demyelination injury. Although robust OPC replication is similarly observed for 7 to 14 days after SCI, many OPCs do not differentiate into mature oligodendrocytes after SCI but are lost to apoptosis, and therefore are incapable of remyelinating axons that survived the primary injury. In this study, we replicated our previous experiment using amiloride, and focused on the fate of OPCs that avoided apoptosis due to the cytoprotective effects of amiloride.

We had previously established that amiloride administration led to a significant increase in NG2-positive OPCs in comparison to the control animals at day 14 [9]. In this follow-up study, we traced OPCs that proliferated between day 1 to 3 after SCI, and demonstrated that these OPCs differentiated into oligodendrocytes (BrdU-APC-positive oligodendrocytes), with a significant difference observed at day 56. Furthermore, we demonstrated that there was significantly higher MBP expression in the amiloride group at days 28 and 56, with a significantly higher myelin sheath thickness/axon diameter in the amiloride group compared to the PBS group at day 28. These results suggest that there was a significant increase in oligodendrocytes brought about by the amiloride treatment, subsequently leading to improved remyelination. The number of BrdU-APC-positive oligodendrocytes was significantly higher in the amiloride group compared to the control group at day 56, while MBP expression was significantly higher at day 28, seemingly indicating a discrepancy. However, since the reduction in secondary injury by amiloride improves the survival of not only newly replicated OPCs, but also resident OPCs and mature oligodendrocytes, it is likely that these cells also provided remyelination, accounting for the early increase in MBP expression.

Both the motor function assessment using the BBB scale and the sensory function assessment using the von Frey test indicated significant improvements at day 14. An improvement at such an early time point suggests a neuroprotective effect of amiloride, possibly through the increased ER stress response that protects it from apoptosis and demyelination, and also because many sodium channel blockers have been shown to exert neuroprotective effects. Based on the differences in functional recovery observed between the amiloride and PBS groups within 56 days after injury, it seems that the therapeutic effects of amiloride are mainly due to the cytoprotective effects of amiloride on resident OPCs and oligodendrocytes, rather than remyelination caused by the OPCs that survived the SCI and proliferated. However, unlike the BBB scores of rats treated with PBS, which seemed to have reached a plateau at approximately six weeks after injury, a plateau was not observed in the BBB scores of the amiloride group during the 56-day study duration, suggesting a continuing trend of functional recovery that may be attributed to remyelination. 

The survival and differentiation of oligodendrocytes, which subsequently remyelinate demyelinated axons, plays an important role in the regenerative processes after SCI. Amiloride inhibits ER stress-induced apoptosis caused by the secondary SCI, and contributes to the survival and differentiation of oligodendrocytes. Therefore, amiloride is a promising candidate drug for the treatment of SCI.
